# Case Report: Isolated and Focal Non-necrotizing Granulomatous Inflammation of Mitral Valves: A Report of Two Cases

**DOI:** 10.3389/fcvm.2021.615707

**Published:** 2021-02-25

**Authors:** Simona Pichler Sekulic, Miroslav Sekulic

**Affiliations:** Department of Pathology and Cell Biology, Columbia University Irving Medical Center, New York, NY, United States

**Keywords:** cardiac valves, non-necrotizing granuloma, mitral valve, prolapse, regurgitation

## Abstract

Cardiac valve inflammation is seen in the setting of autoimmune or infectious processes, and rarely is valvulitis characterized by granulomatous inflammation. We present two patients who underwent surgical repair of prolapsing/regurgitating mitral valves. Excised valve tissue in both cases revealed commonly encountered nodular fibrosis and calcification, however each also revealed an isolated focus of non-necrotizing granulomatous inflammation. Typical implicating etiologies for non-necrotizing granulomatous inflammation were not present for either patient based on clinical history, or radiologic and laboratory data. In a review of 1048 cardiac valves excised at our institution, the finding of non-necrotizing granulomatous inflammation was seen in only the two described cases (prevalence of 0.19%). The description of non-necrotizing granulomatous inflammation within cardiac valves is limited in the literature, and the significance of the detailed isolated and incidental finding is unclear and requiring further investigation.

## Introduction

Granulomatous inflammation within cardiac valves has been described in the setting of autoimmune disease (e.g., sarcoidosis, rheumatoid arthritis, granulomatosis with polyangiitis, Takayasu aortitis) and infectious processes (e.g., *Mycobacterium tuberculosis, Coccidioides immitis, Coxiella burnetii*) (1–7). In these cases, the valves exhibit clinical and echocardiographic evidence of dysfunction illustrating the injurious nature of these processes to the structural integrity of the valves. In contrast, granulomatous inflammation found focally and incidentally within cardiac valves is not well-described in the literature. Herein, we present two patients with mitral valve prolapse and excised mitral valve tissue revealed in each case an isolated focus of non-necrotizing granulomatous inflammation, likely not contributing to valve dysfunction but without a defined etiology.

## Case Descriptions

### Case 1

A 69-year-old Caucasian woman initially presented with shortness of breath, paroxysmal nocturnal dyspnea, and lower extremity edema. Transthoracic echocardiogram showed severe mitral valve regurgitation in the setting of extensive mitral annular calcification, and surgical management was decided upon. A chest X-ray revealed no significant abnormalities including a normal cardiac silhouette. Laboratory testing performed before surgery was notable for a normal complete blood count (CBC). Medical history was notable for hyperlipidemia, gastroesophageal reflux, hypoparathyroidism, osteoarthritis, was a former smoker, and a body mass index (BMI) of 32.3 kg/m^2^. Medication list included bisoprolol, omeprazole, and simvastatin. The patient underwent mitral valve replacement with a bioprosthetic valve.

**Figure 1:**
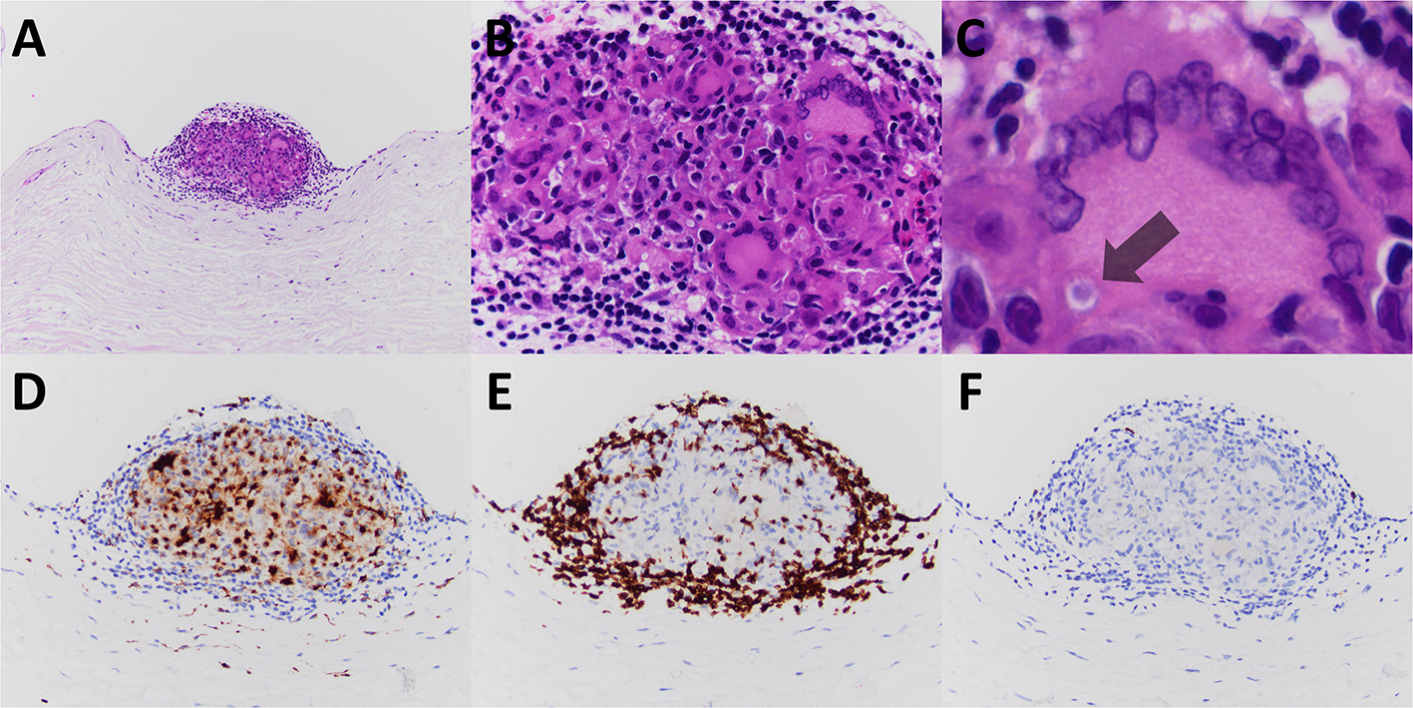
The excised mitral valve of Patient #1 revealed a small well-formed non-necrotizing granuloma, nodular and on the valve surface (**A–C**, hematoxylin and eosin stained paraffin sections). Immunoperoxidase staining for CD68, CD3, and CD20 highlighted the granuloma's constituents of monocyte derived epithelioid histiocytes and multinucleated giant cells **(D)**, T-cell lymphocytes **(E)**, and few B-cell lymphocytes **(F)**, respectively. A multinucleated giant cell contains a rare cytoplasmic inclusion consistent with an asteroid body (**C**, indicated with arrow). Original magnification for **A** at × 100; for **B** at × 400; for **C** at × 600; and for **D–F** at × 200.

Pathologic gross examination of the excised tissue noted leaflet fragments with nodular thickening and calcification. Sections of the valve were submitted for decalcification and processing for light microscopy. Microscopic examination revealed nodular calcification and myxoid degeneration. Additionally, the valve revealed an isolated non-necrotizing granuloma present on the surface of ventricularis layer of the valve (Figure 1). Acid fast bacillus and methenamine silver stains were performed on paraffin sections and were negative for acid fast bacilli and fungal organisms. No polarizable or foreign material was identified in association with the granuloma. Further evidence of granulomatous inflammation was not identified after cutting deeper into the tissue block and after the remainder of the valve specimen was processed for light microscopy.

The postoperative course was unremarkable and the patient was discharged 4 days after surgery. Subsequent to the finding of focal granulomatous inflammation in the mitral valve, a limited work-up followed revealing negative testing for antinuclear antibody, antineutrophil cytoplasmic antibodies (ANCA) screen, interferon-gamma release assay for *Mycobacterium tuberculosis* (T-SPOT TB test), and a blood culture was negative for growth of bacterial or fungal organisms. Follow-up examination 1 month after surgery showed improved functional status with no new clinical developments.

### Case 2

A 68-year-old Caucasian man initially presented with shortness of breath. A transthoracic echocardiogram showed posterior valve leaflet prolapse with severe regurgitation and mitral annular dilatation, and surgical management was decided upon. A chest X-ray revealed a mildly enlarged cardiac silhouette and no other significant abnormalities. Laboratory testing performed before surgery was notable for a normal CBC. Medical history was notable for cerebrovascular accident (status post right internal carotid artery endarterectomy), hyperlipidemia, and a BMI of 29.3 kg/m^2^. Medication list was notable for lisinopril, aspirin, and atorvastatin. The patient underwent mitral valve repair with an annuloplasty band placed.

**Figure 2:**
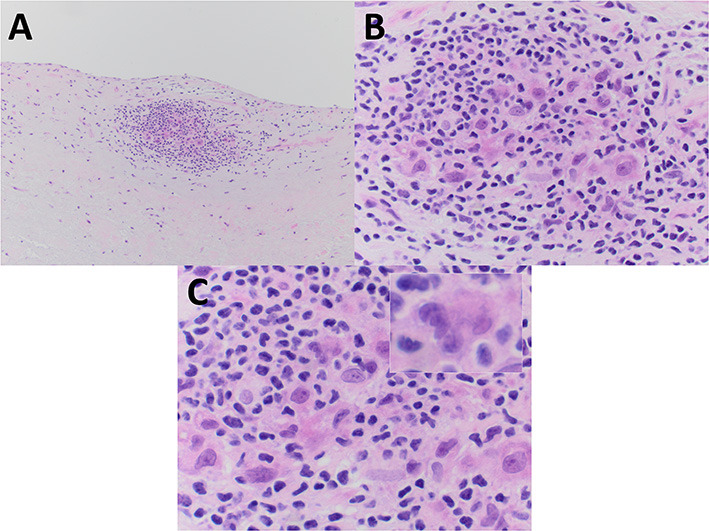
The excised mitral valve of Patient #2 showed a small focus of non-necrotizing granulomatous inflammation, adjacent to the valve surface, and less well-formed as that seen in Patient #1. **(A–C)** Mononuclear inflammatory cells morphologically consistent with lymphocytes surround and partially admix with epithelioid histiocytes and a rare multinucleated giant cell. **(C inset)** All images are from hematoxylin and eosin stained paraffin sections. Original magnification for **A** at × 100; for **B** at × 400; and for **C** and **C** inset at × 600.

Pathologic gross examination of the excised tissue noted leaflet fragments with nodular thickening and focal calcification. Sections of the valve were submitted for decalcification and processing for light microscopy. Microscopic examination revealed nodular calcification and myxoid degeneration. Additionally, the valve revealed an isolated ill-formed (particularly when compared to that seen in Patient #1) non-necrotizing granuloma present on the surface of ventricularis layer of the valve (Figure 2). No polarizable or foreign material was identified in association with the granuloma. Further evidence of granulomatous inflammation was not identified after cutting deeper into the tissue block (the lesion of interest also disappeared upon further sectioning precluding the performing of stains in an effort to evaluate) and after the remainder of the valve specimen was processed for light microscopy.

The postoperative course was unremarkable and the patient was discharged 5 days after surgery. After discussion of the focal finding of granulomatous inflammation in the mitral valve, no further related work-up was decided upon by the clinical team caring for the patient. Follow-up examination 1 month after surgery showed improved functional status with no new clinical developments.

## Discussion

Granulomatous inflammation of the cardiac valves can be identified in the settings of autoimmune and infectious processes. In the literature to date, examples of granulomatous inflammation involving cardiac valves show evidence of valvular dysfunction with regurgitation. More subtle and non-destructive forms of valve involvement by granulomatous inflammation has not been well-described, but in this report two examples are presented of mitral valves excised in the setting of regurgitation reveal minute and focal non-necrotizing granulomas which likely contribute in no significant manner to valve structural integrity and function are described.

Granulomatous valvulitis has been described previously in few cases of patients with varying autoimmune disease.

While heart wall involvement in cardiac sarcoidosis can be prominent, valvular involvement is less commonly described but when present has been shown to result in regurgitation (6). In the setting of rheumatoid arthritis, all cardiac valves have been described to be involved by non-necrotizing granulomatous inflammation with features shared with rheumatoid nodule formation of other tissue sites (3). Granulomatosis with polyangiits (ANCA-associated vasculitis) has also been shown to have necrotizing granulomatous inflammation of the cardiac valves (4). An unusual case of aortic valve regurgitation was determined upon excision to have aortic valve involvement with giant cell granulomatous valvulitis extending from the aorta in a patient with Takayasu aortitis (5). As opposed to our presented two patients, the described autoimmune associated inflammation significantly contributed to valvular dysfunction and regurgitation. Neither of our two patients had clinical histories or examination findings to suggest underlying systemic autoimmune disease, and Patient #1's limited work-up revealed no supportive evidence *via* screening serologic assays. Additionally, chest X-rays did not reveal pulmonary changes or lymphadenopathy suggestive of sarcoidosis.

Infectious etiologies have been implicated in the development of granulomatous valvulitis, including both bacterial (*Mycobacterium tuberculosis* and *Coxiella burnetii*) and fungal (*Coccidioides immitis*) organisms (1, 2, 7). As opposed to our presented two patients, the previously published cases of infectious granulomatous valvulitis exhibited significant valvular dysfunction and regurgitation. Neither of our two patients had clinical histories or examination findings (both afebrile at all points of care) to suggest an infectious process, and Patient #1's limited work-up of a T-SPOT TB test and blood cultures were negative.

As the cardiac valves are contiguous with the heart wall and exposed to the same passing blood flow, etiologic considerations of granulomatous myocarditis should also potentially be extrapolated for granulomatous valvulitis. Granulomatous inflammation (excluding pathologies already described to involve the valves, *vide supra*) of the myocardium is present in autoimmune disease (e.g., rheumatic fever, sarcoidosis, Behçet's disease), giant cell myocarditis, drug-related hypersensitivity reaction, metabolic disorders (e.g. Farber's disease/lipogranulomatosis, gout, tophi, oxalosis), and as a reaction to foreign and sometimes iatrogenically introduced material (e.g., suture material, oxidized cellulose, starch granules) (8–12). In the presented cases there was no evidence of foreign material associated with the focal non-necrotizing granulomas. As already noted the patients did not exhibit evidence of autoimmune disease or hypersensitivity reaction (normal CBC without evidence of eosinophilia). With respect to circulating foreign material, Patient #1 had no prior surgical history and although Patient #2 was status post endarterectomy the potential introduction of foreign material into the internal carotid artery blood stream would not result in it reaching the left side of the heart. Neither patient had history of intravenous (IV) drug use, although Patient #2 likely received IV agents during the endarterectomy.

A retrospective review of the electronic pathology database at our institution was searched for sampled native cardiac valves not involved by acute inflammatory processes (i.e., acute bacterial endocarditis, non-bacterial thrombotic endocarditis et al.), and those not having been previously intervened/repaired *via* surgery or endovascular techniques. The most recent 1,048 valves (this includes all four cardiac valve types) were thus identified and reviewed, which were provided from either surgical excision or autopsy. The finding of non-necrotizing granulomatous inflammation was present only in the two described cases (prevalence of 0.19%, with a 95% confidence interval, of 0.05–0.69%).

In summary, we have presented two cases of non-necrotizing granulomatous inflammation focally involving mitral valves. Clinical and laboratory data for both patients do not provide an etiology to explain the incidentally discovered finding and as such represents an idiopathic process of likely minimal significance. Further investigation into such focal non-necrotizing granulomatous inflammation of cardiac valves is required to determine the significance (if any) of the very focal and limited finding.

## Data Availability Statement

The original contributions presented in the study are included in the article/supplementary materials, further inquiries can be directed to the corresponding author/s.

## Ethics Statement

The studies involving human participants were reviewed and approved by Columbia University Medical Center. The patients/participants provided their written informed consent to participate in this study. Written informed consent was obtained from the individual(s) for the publication of any potentially identifiable images or data included in this article.

## Author Contributions

All authors listed have made a substantial, direct and intellectual contribution to the work, and approved it for publication.

## Conflict of Interest

The authors declare that the research was conducted in the absence of any commercial or financial relationships that could be construed as a potential conflict of interest.
